# Binding of Tetracycline and Chlortetracycline to the Enzyme Trypsin: Spectroscopic and Molecular Modeling Investigations

**DOI:** 10.1371/journal.pone.0028361

**Published:** 2011-12-19

**Authors:** Zhenxing Chi, Rutao Liu, Hongxu Yang, Hengmei Shen, Jing Wang

**Affiliations:** Shandong Provincial Key Laboratory of Water Pollution Control and Resource Reuse, School of Environmental Science and Engineering, Shandong University, China-America CRC for Environment and Health, Shandong Province, Jinan, People's Republic of China; University of Cambridge, United Kingdom

## Abstract

Tetracycline (TC) and chlortetracycline (CTC) are common members of the widely used veterinary drug tetracyclines, the residue of which in the environment can enter human body, being potentially harmful. In this study, we establish a new strategy to probe the binding modes of TC and CTC with trypsin based on spectroscopic and computational modeling methods. Both TC and CTC can interact with trypsin with one binding site to form trypsin-TC (CTC) complex, mainly through van der Waals' interactions and hydrogen bonds with the affinity order: TC>CTC. The bound TC (CTC) can result in inhibition of trypsin activity with the inhibition order: CTC>TC. The secondary structure and the microenvironment of the tryptophan residues of trypsin were also changed. However, the effect of CTC on the secondary structure content of trypsin was contrary to that of TC. Both the molecular docking study and the trypsin activity experiment revealed that TC bound into S1 binding pocket, competitively inhibiting the enzyme activity, and CTC was a non-competitive inhibitor which bound to a non-active site of trypsin, different from TC due to the Cl atom on the benzene ring of CTC which hinders CTC entering into the S1 binding pocket. CTC does not hinder the binding of the enzyme substrate, but the CTC-trypsin-substrate ternary complex can not further decompose into the product. The work provides basic data for clarifying the binding mechanisms of TC (CTC) with trypsin and can help to comprehensively understanding of the enzyme toxicity of different members of tetracyclines *in vivo*.

## Introduction

**Figure 1:**
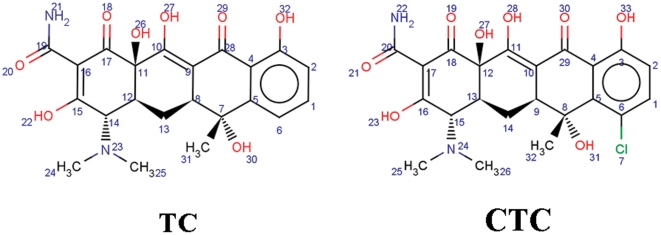
Molecular structure of TC and CTC (with atom numbers).

Tetracycline (TC) and chlortetracycline (CTC) (structure with atom numbers shown in Fig. 1) are common members of tetracyclines, widely used for disease control and as feed additive in livestock for several decades due to their great therapeutic values [1]. Similar to other antibiotics, they are excreted mostly as the parent compound, representing 50–80% of the applied dose [2]. The excreted tetracyclines can enter soils, surface and ground water, being a potential risk to human health [3]. The toxicity of tetracyclines residues in the environment including animal food [4], soils [5], and surface and groundwater [3], has attracted widespread attention [6].

The water-soluble globular protein, trypsin (EC 3.4.21.4, structure shown in Figure S1) is a proteolytic enzyme that is excreted by the pancreas into the small intestine and takes part in the digestion of food proteins and other biological processes [7]. However, intake of any contaminants is likely to affect the activity of the enzyme *in vivo*. Tetracyclines interfere with processes of secretion as well as synthesis of pancreatic protein in pigeons [8], has effect on the secretion kinetics of the rat exocrine pancreas and can lead to the decrease of trypsin level in male wistar rats [9], [10]. The fact indicates that tetracyclines can enter the pancrea. So, they have potential to interact with trypsin to affect the structure and function of trypsin [11]. We have studied the binding of oxytetracycline (belonging to tetracyclines) with trypsin [12]. However, the interaction mechanism of the other common tetracyclines (TC and CTC) with trypsin has not been studied previously. In this work, the binding mechanisms of TC and CTC with trypsin were investigated by multispectroscopic techniques and molecular modeling methods. The study is helpful for comprehensive understanding of the enzyme toxicity of different members of tetracyclines *in vivo* and their toxicity assessment in the environment.

## Results and Discussion

### Characterization of the Binding Interactions of TC (CTC) with Trypsin by Fluorescence Measurements Based on the Elimination of the Inner Filter Effects

In this study, we eliminated the inner filter effect for all of the fluorescence and synchronous fluorescence results to obtain accurate data. The fluorescence intensity was corrected using the eq 1.

#### Fluorescence spectra

**Figure 2:**
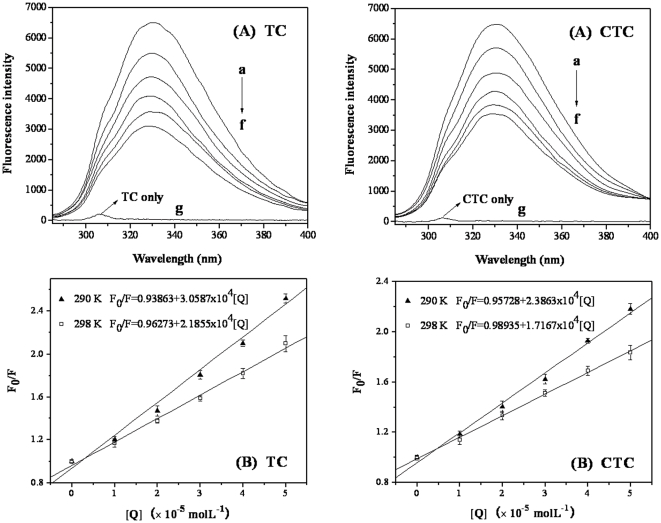
(A) Effect of TC (CTC) on trypsin fluorescence (corrected). (B) Stern-Volmer plots for the quenching of trypsin by TC and CTC at different temperatures (corrected). Data represent the mean ± SD of three independent experiments. **Conditions:** (A) trypsin (5×10^−6^ mol L^−1^) with different concentrations of TC and CTC (×10^−5^ mol L^−1^, a, 0; b, 1; c, 2; d, 3; e, 4; f, 5); g: TC (CTC) only, concentration 5×10^−5^ mol L^−1^; pH 7.6; T = 298 K. (B) trypsin concentration: 5×10^−6^ mol L^−1^; pH 7.6.

The fluorescence intensity (F) of trypsin decreased regularly with increasing TC (CTC) concentrations (Figure 2A). Furthermore, a small blue shift was observed for the emission wavelengths, which suggests that the fluorescence chromophore of trypsin was placed in a more hydrophobic environment after the addition of TC (CTC) [13].

The fluorescence quenching data were analyzed according to the Stern-Volmer eq 2 to determine the quenching mechanism which can investigate whether TC (CTC) interact with trypsin to form trypsin-TC (CTC) complex. The Stern-Volmer plots for the quenching of trypsin by TC (CTC) at two different temperatures after correction are shown in Figure 2B. The *K*_SV_ and *k*_q_ at two different temperatures are listed in Table 1. The *K*_SV_ values decreased with increasing temperature and *k*_q_ was greater than 2.0×10^10^ Lmol^−1^ s^−1^ [14], [15]. These results indicate that the quenching was not initiated from dynamic collision but from the formation of a complex.

**Table 1:** Stern-Volmer quenching constants for the interaction of TC (CTC) with trypsin at different temperatures.

T (K)		*K*_SV_ (×10^5^ L mol^−1^)	*k*_q_ (×10^12^ Lmol^−1^ s^−1^)	R^a^	S.D.^b^
TC	290	0.30587	3.0587	0.99518	0.06302
	298	0.21855	2.1855	0.99701	0.03544
CTC	290	0.23863	2.3863	0.99674	0.04041
	298	0.17167	1.7167	0.999	0.01604

a R is the correlation coefficient.

b S.D. is the standard deviation for the K_SV_ values.

#### Binding parameters

The quenching mechanism was determined to be static quenching, so the binding constant (*K*_a_) and the number of binding sites (n) can be calculated according to eq 3. A plot of log[(*F*_0_-*F*)/*F*] vs log[TC (CTC)] yields log*K*_a_ as the intercept on the *y* axis and *n* as the slope (Figure S2). Table 2 shows the calculated *K*_a_ and n. The number of binding sites, n, is approximately 1, indicating that there is one binding site in trypsin for TC (CTC) during their interaction. The affinity order is as follows: TC>CTC.

**Table 2:** Binding constants and relative thermodynamic parameters of the TC (CTC)-trypsin system.

T (K)		K_a_(×10^5^ L mol^−1^)	n	R^a^	ΔH°(kJmol^−1^)	ΔS°(Jmol^−1^ K^−1^)	ΔG°(kJmol^−1^)
TC	290	1.8479	1.18	0.99935	−64.662	−122.32	−29.138
	298	0.8995	1.14	0.99976			−28.262
CTC	290	1.0383	1.15	0.99936	−52.64	−85.486	−27.849
	298	0.5778	1.12	0.9978			−27.165

a R is the correlation coefficient for the *K*_a_ values.

#### Thermodynamic parameters and binding forces

The acting forces between small organic molecules and biomolecules include hydrogen bonds, van der Waals interactions, electrostatic forces and hydrophobic interaction forces. Because the temperature effect is very small, the interaction enthalpy change (Δ*H*°) can be regarded as a constant if the temperature range is not too wide. The enthalpy change (Δ*H*°), free-energy change (Δ*G*°) and the entropy change (Δ*S*°) for the interaction between TC (CTC) and trypsin were calculated according to the van't Hoff equation (eq 4) and thermodynamic equation (eq 5) (Table 2). The negative Δ*H*° and Δ*S*° indicated that van der Waals interactions and hydrogen bonds play the major role during the interaction. In addition, the negative sign of Δ*G* ^0^ indicates the binding of TC (CTC) with trypsin is spontaneous [14].

#### Energy transfer between TC (CTC) and trypsin

The overlap of the absorption spectrum of TC (CTC) with the fluorescence emission spectrum of trypsin is shown in Figure S3. According to Förster's nonradiative energy transfer theory [16], the energy transfer will happen under the conditions: (i) the donor can produce fluorescence light; (ii) the absorption spectrum of the receptor overlaps enough with the donor's fluorescence emission spectrum; (iii) the distance between the donor and the acceptor is less than 8 nm.

It has been reported for trypsin that, *K* ^2^ = 2/3, *n* = 1.336 and Φ = 0.118 [17]. *J* in eq 8 was evaluated by integrating the UV absorption and fluorescence emission spectra (Figure S3). Based on these data and eq 6–9, the overlap integral *J*, *R*_0_, *E*, and *r* can be evaluated (Table 3). The distance between TC (CTC) and the tryptophan residues in trypsin after interaction is lower than 8 nm. These accords with conditions of Förster's nonradiative energy transfer theory, indicating again the static quenching interaction (forming a complex) between TC (CTC) and trypsin.

**Table 3:** Energy transfer parameters between TC (CTC) and trypsin.

Parameters	Overlap integral J (cm^3^ L mol^−1^)	Efficiency of transfer E	Critical distance R_0_ (nm)	Energy transfer distance r (nm)
TC	1.603×10^−14^	0.0672	2.65	4.11
CTC	1.462×10^−14^	0.0699	2.61	4.02

In summary, Both TC and CTC can interact with trypsin through van der Waals' interactions and hydrogen bonds with one binding site to form a complex. The binding process is spontaneous.

### Effect of TC (CTC) on Trypsin Activity

**Figure 3:**
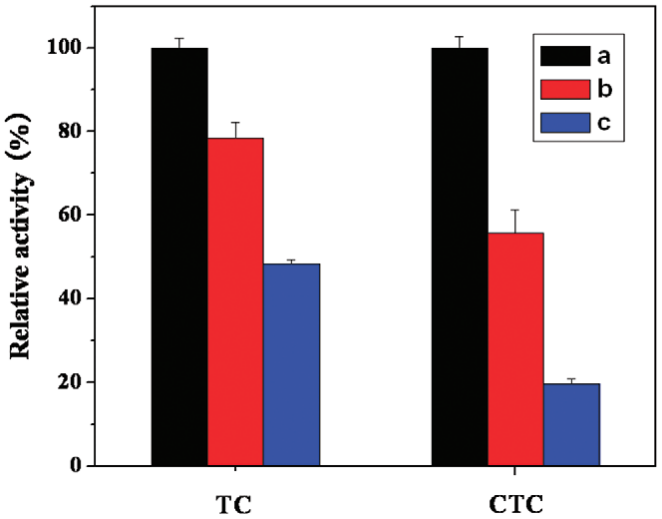
Effect of TC and CTC on the activity of trypsin. Data represent the mean ± SD of three independent experiments. **Conditions:** trypsin (1.67×10^−6^ mol L^−1^) with different concentrations of TC and CTC (×10^−5^ mol L^−1^, a, 0; b, 10; c, 20); pH 7.6; T = 298 K.

The effects of different concentrations of TC (CTC) on the activity of trypsin are presented in Figure 3. The trypsin activity decreased with increasing TC (CTC) concentrations. At a molar ratio of 120∶1 (TC (CTC) to trypsin), the activity of trypsin decreased to only 48.3 and 19.6% of the initial level for TC and CTC, respectively. Both TC and CTC can inhibit trypsin activity with the inhibition order as follows: CTC>TC.

**Figure 4:**
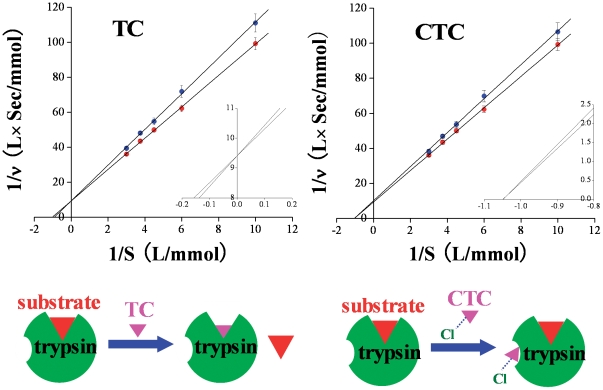
Lineweaver-Burk plots of trypsin in the presence (blue circle) and absence (red circle) of TC (CTC), and the schematic diagrams for the binding of TC and CTC with trypsin. Data represent the mean ± SD of five independent experiments. **Conditions:** trypsin concentration: 1.67×10^−6^ mol L^−1^; TC and CTC concentration: 3.33×10^−5^ mol L^−1^; pH 7.6.

To confirm the inhibition mode, the lineweaver-burk plots of trypsin with and without TC and CTC were drawn (Figure 4). The lineweaver-burk plots for TC intersect on the vertical axis, showing a typical feature of competitive inhibition [18]. However, the lineweaver-burk plots with and without CTC intersect on the abscissa axis, which is the typical noncompetitive inhibition characteristic. The inhibition mechanisms were illustrated in Figure 4. TC binds to the active site of trypsin and hinders the binding of the enzyme substrate, leading to the enzyme inhibition. Although CTC binds to a non-active site of trypsin which does not hinder the binding of the enzyme substrate, the CTC-trypsin-substrate ternary complex can not further decompose into the product.

### Computational Modeling of the Trypsin-TC (CTC) Complex

**Figure 5:**
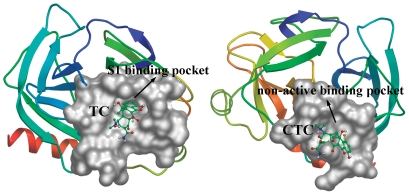
The binding mode between TC (CTC) and trypsin. Trypsin is shown in cartoon. The interacting side chains of trypsin are displayed in surface mode. TC and CTC are represented using balls and stick. The atoms of TC and CTC are color-coded as follows: O, red; N: blue; C, green; H, white.

**Figure 6:**
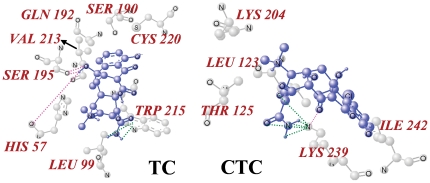
The molecular modeling of interaction between TC (CTC) and trypsin. The atoms of TC and CTC are marked with blue and the atoms of amino acid residues of trypsin are labeled with gray. The hydrogen bonds between TC (CTC) and trypsin are indicated by pink dashed line. The van der Waals interactions are illustrated with green dashed line.

Both TC and CTC can interact with trypsin with one binding site to form trypsin-TC (CTC) complex. So we employed the molecule docking method to find the specific binding site of TC (CTC) on trypsin. The crystal structure of trypsin was taken from the Protein Data Bank (entry PDB code 2ZQ1). The best energy ranked results are shown in Figure 5 and Figure 6.

Trypsin is serine protease, with the catalytic triad composed of His-57, Asp-102 and Ser-195, and the primary substrate-binding pocket (S1 binding pocket) formed by residues 189–195, 214–220 and 225–228 [19], [20]. It can be shown in Figure 5 that TC binds into the S1 binding pocket, occupying the active site of the enzyme substrate. Hence, the trypsin activity is competitively inhibited by TC [21]. However, CTC does not bind into the S1 binding pocket. So the trypsin activity is noncompetitively inhibited. The computational modeling investigation further confirms the conclusions of the trypsin activity experiment.

For TC, hydrogen bonds exist between the hydrogen atom on the oxygen atom at position 30 of TC and the oxygen atoms on HIS 57 and SER 195, the oxygen atom at position 30 of TC and that on SER 195 (Figure 6). There are van der Waals interactions between the two hydrogen atoms on the nitrogen atom at position 21 of TC and the residue TRP 215. For CTC, there are hydrogen bonds between the oxygen atom at position 28 of CTC and the nitrogen atom on LYS 239 (Figure 6). The van der Waals interactions exist between the nitrogen atom on LYS 239 and the oxygen atoms at position 21 and position 23, the nitrogen atom at position 22 and the two hydrogen atoms on the nitrogen atom at position 22 of CTC. In addition, the modeled structure showed that hydrophobic interactions (TC with VAL 213, CTC with ILE 242) and other forces (TC with HIS 57, LEU 99, SER 190, GLN 192, SER 195, TRP 215 and CYS 220, and CTC with LEU 123, THR 125, LYS 204, LYS 239 and ILE 242) are also present. However, the hydrogen bonds and van der Waals interactions play a major role in the binding of TC and CTC to trypsin, in agreement with our conclusion of thermodynamic analysis (negative Δ*H*° and Δ*S*°).

From the docking result of TC with trypsin (Figure 5), we found that it is the benzene ring that first enters into the S1 binding pocket during its interaction with the enzyme. The molecular structure of CTC is the same as that of TC except the chlorine atom on the benzene ring (Figure 1). The Cl atom hinders CTC entering into the S1 binding pocket, resulting in its different binding site on trypsin and enzyme activity inhibition mechanism from TC.

### Investigation of Trypsin Conformation Changes

#### UV-Vis absorption spectra studies

**Figure 7:**
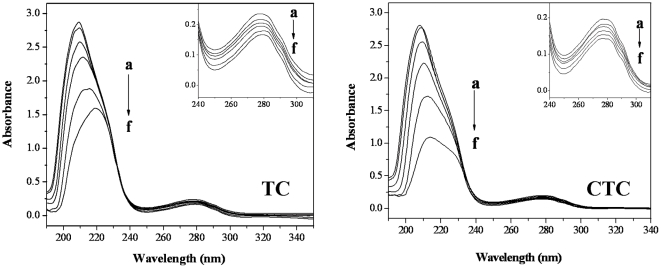
UV-vis spectra investigation. **Conditions:** trypsin (4×10^−5^ mol L^−1^) with different concentrations of TC and CTC (×10^−5^ mol L^−1^, a, 0; b, 2; c, 6; d, 10; e, 15; f, 20) (vs the same concentration of TC (CTC) solution); pH 7.6; T = 298 K.

UV-vis absorption spectroscopy technique can be used to explore the structural changes of protein and to investigate protein-ligand complex formation. The UV-vis absorption spectra of trypsin in the presence and absence of TC (CTC) are shown in Figure 7. Trypsin has two absorption peaks. The strong absorption peak at about 206 nm reflects the framework conformation of the protein [22]. The weak absorption peak at about 274 nm appears to be due to the aromatic amino acids (Trp, Tyr and Phe) [23]. With gradual addition of TC (CTC) to trypsin solution, the intensity of the peak at 206 nm decreases and red shifts and the intensity of the peak at 274 nm also decreases. The results indicate that the interactions between TC (CTC) and trypsin leads to the loosening and unfolding of the protein skeleton and increases the hydrophobicity of the microenvironment of the aromatic amino acid residues [24].

#### Synchronous fluorescence

Synchronous fluorescence spectroscopy can give information about the molecular environment in the vicinity of chromophores. The spectrum is obtained through the simultaneous scanning of the excitation and emission monochromators while maintaining a constant wavelength interval between them. When the wavelength intervals (**Δλ**) are stabilized at 15 nm or 60 nm, the synchronous fluorescence gives the characteristic information of tyrosine residues or tryptophan residues, respectively [25].

**Figure 8:**
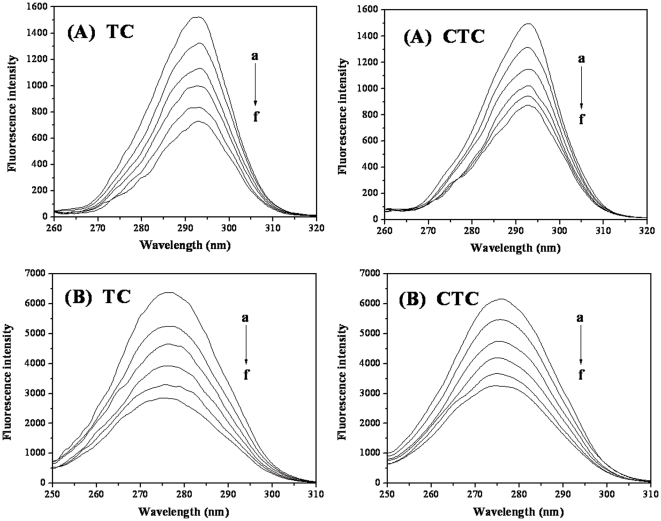
Synchronous fluorescence spectra of trypsin (corrected). (A) Δλ = 15 nm and (B) Δλ = 60 nm. **Conditions:** trypsin (5×10^−6^ mol L^−1^) with different concentrations of TC and CTC (×10^−5^ mol L^−1^, a, 0; b, 1; c, 2; d, 3; e, 4; f, 5); pH 7.6; T = 298 K.

The synchronous fluorescence spectra of trypsin with various amounts of TC (CTC) in Figure 8A show that the emission peaks do not shift over the investigated concentration range, which indicates that TC (CTC) have little effect on the microenvironment of the tyrosine residues in trypsin. In Figure 8B, the emission maximum of the tryptophan residue shows a slight blue shift (from 276 to 275, 274.8 nm for TC and CTC, respectively) which indicates that the conformation of trypsin was changed such that the polarity around the tryptophan residues decreased and the hydrophobicity was increased [26].

#### Circular dichroism

**Figure 9:**
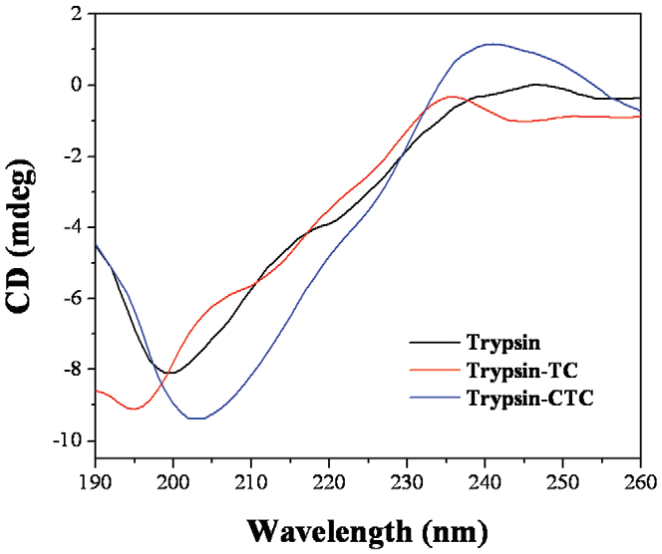
CD spectra of trypsin and the trypsin-TC (CTC) system at room temperature. **Conditions:** trypsin (5×10^−5^ mol L^−1^) with 0 or 5×10^−5^ mol L^−1^ TC (CTC); pH 7.6.

To ascertain the possible influence of TC (CTC) binding on the secondary structure of trypsin, CD measurements were performed in the presence of different TC (CTC) concentrations (Figure 9). The estimates for the secondary structural elements found by the Jasco secondary structure manager software are listed in Table 4.

**Table 4:** The effects of TC (CTC) on the percentage of secondary structural elements in trypsin.

Molar ratio(trypsin to TC (CTC))	Secondary structural elements in trypsin			
	Helix (±0.1%)	Beta (±2%)	Turn (±1%)	Random (±1%)
1∶0	1.2	49.5	8.6	40.8
1∶1 TC	1.7	42.6	15.6	40.1
1∶1 CTC	0.5	54.5	4.8	40.2

The CD spectrum of trypsin exhibits a negative band in the ultraviolet region at about 199 nm. The calculated secondary structure content of trypsin was 1.2% α-helix, 49.5% β-pleated sheet, 8.6% β-turn and 40.8% random coil. With the addition of TC (CTC), the intensity of the negative peak increased with blue shift for TC. However, the negative peak increased with red shift for CTC. At trypsin/TC ratio of 1∶1, the α-helix increased by 0.5%, the β-pleated sheet decreased by 6.9%, and the β-turn increased by 7%. For CTC, the α-helix decreased by 0.7%, the β-pleated sheet increased by 5%, and the β-turn decreased by 3.8%, having reverse trend from that of TC. The bound CTC has opposite impact on the secondary structure content of trypsin compared to TC, because of the different binding site of TC and CTC on trypsin, in good accordance with the conclusion of the trypsin activity experiment and the molecule docking investigation.

As mentioned above, the binding of TC (CTC) can lead to the loosening and unfolding of the protein skeleton and increases the hydrophobicity of the microenvironment of the tryptophan residues of trypsin. The α-helix and the β-turn of the secondary structure of trypsin increased and the β-pleated sheet decreased because of bound TC. However, the bound CTC has reverse impact on the secondary structure content of trypsin compared to TC.

### Conclusions

In this paper, we simulated and compared the interaction of the widely used veterinary drug tetracyclines (TC and CTC) with trypsin. The experimental results indicate that both TC and CTC can interact with trypsin with one binding site mainly through van der Waals interactions and hydrogen bonds with the affinity order: TC>CTC. TC bound into S1 binding pocket, competitively inhibiting the enzyme activity, and CTC was a non-competitive inhibitor which bound to a non-active site of trypsin, different from TC because of the Cl atom on the benzene ring of CTC which hinders CTC entering into the S1 binding pocket. CTC does not hinder the binding of the enzyme substrate, but the CTC-trypsin-substrate ternary complex can not further decompose into the product. The binding of TC (CTC) can also change the secondary structure and the microenvironment of the tryptophan residues of trypsin. However, CTC has opposite effect on the secondary structure content of trypsin from TC because of their different binding sites on trypsin. The established research route combing spectroscopic techniques and the molecular modeling method in the research is worth spreading to explore the binding mechanisms of other small organic pollutants and drugs.

## Materials and Methods

### Reagents

Trypsin (from bovine pancreas, Amresco) was dissolved in ultrapure water to form a 5×10^−4^ mol L^−1^ solution, then preserved at 0–4°C and diluted as required. We prepared stock solutions of TC (1.0×10^−3^ mol L^−1^) and CTC (1.0×10^−3^ mol L^−1^) by dissolving 0.0481 g tetracycline hydrochloride (Sigma) and 0.0515 g chlortetracycline hydrochloride (Amresco) in 100 mL of water, respectively.

BAEE (N-α-benzoyl-L-arginine ethyl ester, from Sinopharm Chemical Reagent Co., Ltd., BR) was dissolved in ultrapure water to form a 1.0×10^−2^ mol L^−1^ solution.

Phosphate buffer (0.2 mol L^−1^, mixture of NaH_2_PO_4_·2H_2_O and Na_2_HPO_4_·12H_2_O, pH 7.6) was used to control pH. NaH_2_PO_4_·2H_2_O and Na_2_HPO_4_·12H_2_O were of analytical reagent grade, obtained from Tianjin Damao Chemical Reagent Factory.

### Apparatus and Methods

All fluorescence spectra were recorded on an F-4600 spectrofluorimeter (Hitachi, Japan). The excitation and emission slit widths were set at 5.0 nm. The scan speed was 1200 nm/min. PMT (photo multiplier tube) voltage was 800 V.

UV-visible absorption spectra were measured on a UV-2450 spectrophotometer (Shimadzu, Kyoto, Japan). CD spectra were recorded on a J-810 CD spectrometer (JASCO). The pH measurements were made with a pHs-3C acidity meter (Pengshun, Shanghai, China).

#### CD measurements

CD spectra were collected from 190 to 260 at 0.2 nm intervals on a JASCO J-810 CD spectrometer. Three scans were made and averaged for each CD spectrum. The protein conformation was computed using Yang.jwr software [27].

#### Fluorescence measurements

The fluorescence measurements were carried out as follows: to each of a series of 10 mL test tubes, 1.0 mL of 0.2 mol L^−1^ phosphate buffer (pH 7.6) and 1.0 mL of 5×10^−5^ mol L^−1^ trypsin were added, and then different amounts of 1.00×10^−3^ mol/L stock solution of TC and CTC were added. The fluorescence spectra were then measured (excitation at 278 nm and emission wavelengths of 285–450 nm). The synchronous fluorescence spectra were measured at λ_ex_ = 250 nm, Δλ = 15 nm and Δλ = 60 nm.

To eliminate the inner filter effects of protein and ligand, absorbance measurements were performed at excitation and emission wavelengths of the fluorescence measurements. The fluorescence intensity was corrected using the equation [28]:
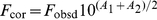
(1)where *F*_cor_ and *F*_obsd_ are the corrected and observed fluorescence intensities, respectively; whereas *A*_1_ and *A*_2_ are the sum of the absorbance of protein and ligand at the excitation and emission wavelengths, respectively.

To confirm the quenching mechanism, the fluorescence quenching data were analyzed according to the Stern-Volmer equation [29]:
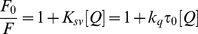
(2)where *F*_0_ and *F* are the fluorescence intensities in the absence and presence of the quencher, respectively. *K*_SV_ is the Stern-Volmer quenching constant, [*Q*] is the concentration of the quencher, *k*_q_ is the quenching rate constant of the biological macromolecule and *τ*_0_ is the fluorescence lifetime in the absence of quencher.

#### Binding parameters

For the static quenching interaction, when small molecules bind independently to a set of equivalent sites on a macromolecule, the binding constant (*K*_a_) and the number of binding sites (*n*) can be determined by the following equation [30]:
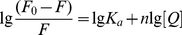
(3)where *F*_0_, *F* and [*Q*] are the same as in eq 2, *K*_a_ is the binding constant, and n is the number of binding sites per trypsin molecule.

#### Thermodynamic parameters

If the enthalpy change (Δ*H*°) does not vary significantly over the temperature range studied, the enthalpy change (Δ*H*°), free-energy change (Δ*G*°) and the entropy change (Δ*S*°) can be calculated based on the van't Hoff equation (eq 4) and thermodynamic equation (eq 5):
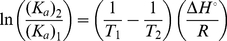
(4)

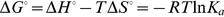
(5)where (*K_a_*)_1_ and (*K_a_*)_2_ are the binding constants at *T*_1_ and *T*_2_ and *R* is the universal gas constant.

#### Energy transfer calculation

According to the Förster's nonradiation energy transfer theory, the parameters related to energy transfer can be calculated based on the equations as follows.

The energy transfer effect is related not only to the distance between the acceptor and the donor but also to the critical energy transfer distance, that is:
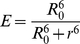
(6)where *r* is the distance between the acceptor and the donor, *R*_0_ is the critical distance when the transfer efficiency is 50%, which can be calculated by:
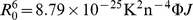
(7)where K^2^ is the spatial orientation factor of the dipole, n is the refractive index of the medium, Φ is the fluorescence quantum yield of the donor, and *J* is the overlap integral of the fluorescence emission spectrum of the donor and the absorption spectrum of the acceptor,
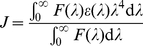
(8)where *F*(*λ*) is the fluorescence intensity of the fluorescence donor at wavelength *λ* and *ε*(*λ*) is the molar absorption coefficient of the acceptor at wavelength *λ*. The energy transfer efficiency is given by
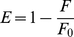
(9)where *F*_0_ and *F* are the same in eq 2.

#### Trypsin activity determination

The activity of trypsin was measured using BAEE as the substrate. Trypsin can catalyze BAEE into N-benzoyl-L-arginine. The UV absorption intensity of BAEE is far weaker than that of N-benzoyl-L-arginine. The enzyme activity was obtained based on the increase in absorbance at 253 nm in 2.0×10^−2^ mol L^−1^ phosphate buffer (mixture of NaH_2_PO_4_·2H_2_O and Na_2_HPO_4_·12H_2_O, pH 7.6) containing 3.0×10^−4^ mol L^−1^ BAEE before and after the addition of trypsin (concentration given in the legend of figure 3 and 4) [31].

The velocity of the enzymatic reaction (ν, mmol L^−1^ S^−1^) was calculated using the following equation:
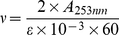
(10)where *A_253 nm_* is the absorbance of the reaction system within 0.5 min, deducted the absorbance of TC and CTC when they exist in the system. *ε* is the molar extinction coefficient of BAEE.

#### Molecular modeling study

Docking calculations were carried out using DockingServer [32]. The MMFF94 force field was used for energy minimization of the ligand molecule using DockingServer [33]. Gasteiger partial charges were added to the ligand atoms. Nonpolar hydrogen atoms were merged, and rotatable bonds were defined.

Docking calculations were carried out on a trypsin protein model (PDB code 2ZQ1). Essential hydrogen atoms, Kollman united atom type charges, and solvation parameters were added with the aid of AutoDock tools [34]. Affinity (grid) maps of 100×100×100 Å grid points and 0.375 Å spacing were generated using the Autogrid program [34]. The AutoDock parameter set and distance-dependent dielectric functions were used in the calculation of the van der Waals and the electrostatic terms, respectively.

Docking simulations were performed using the Lamarckian genetic algorithm (LGA) and the Solis & Wets local search method [35]. Initial positions, orientations, and torsions of the ligand molecules were set randomly. Each run of the docking experiment was set to terminate after a maximum of 250000 energy evaluations. The population size was set to 150. During the search, a translational step of 0.2 Å, and quaternion and torsion steps of 5 were applied.

## Supporting Information

Figure S1 **Molecular structure of trypsin (PDB code 2ZQ1).** Different types of the secondary structure of trypsin are colour-coded as follows: α-helix: magenta, β-pleated sheet: yellow, β-turn: aquamarine, random coil: white.
(TIF)

Figure S2 **Plot of log [(** ***F*** **_0_-** ***F*** **)/** ***F*** **] vs log [TC (CTC)] for the binding of TC and CTC to trypsin at various temperatures.**
(TIF)

Figure S3 **Overlap of the absorption spectrum of TC and CTC with fluorescence emission spectrum of trypsin (corrected).** **Conditions:** Curve a: the absorption spectrum of TC (CTC); Curve b: the fluorescence emission spectrum of trypsin. The concentration of both TC (CTC) and trypsin are 5×10^−6^ mol L^−1^.
(TIF)
