# Supplementary figures and images for: Binding of Tetracycline and Chlortetracycline to the Enzyme Trypsin: Spectroscopic and Molecular Modeling Investigations
Source: PLoS One. 2011 Dec 19;6(12):e28361. doi: 10.1371/journal.pone.0028361 (PMC3242759; doi:10.1371/journal.pone.0028361)

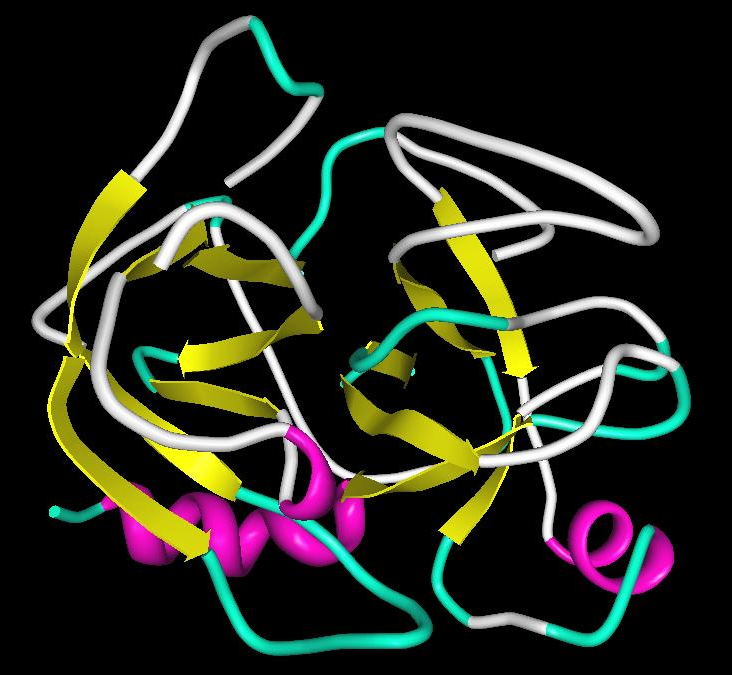

Supplement: Figure S1 — Molecular structure of trypsin (PDB code 2ZQ1). Different types of the secondary structure of trypsin are colour-coded as follows: α-helix: magenta, β-pleated sheet: yellow, β-turn: aquamarine, random coil: white. (TIF) [file pone.0028361.s001.tif]

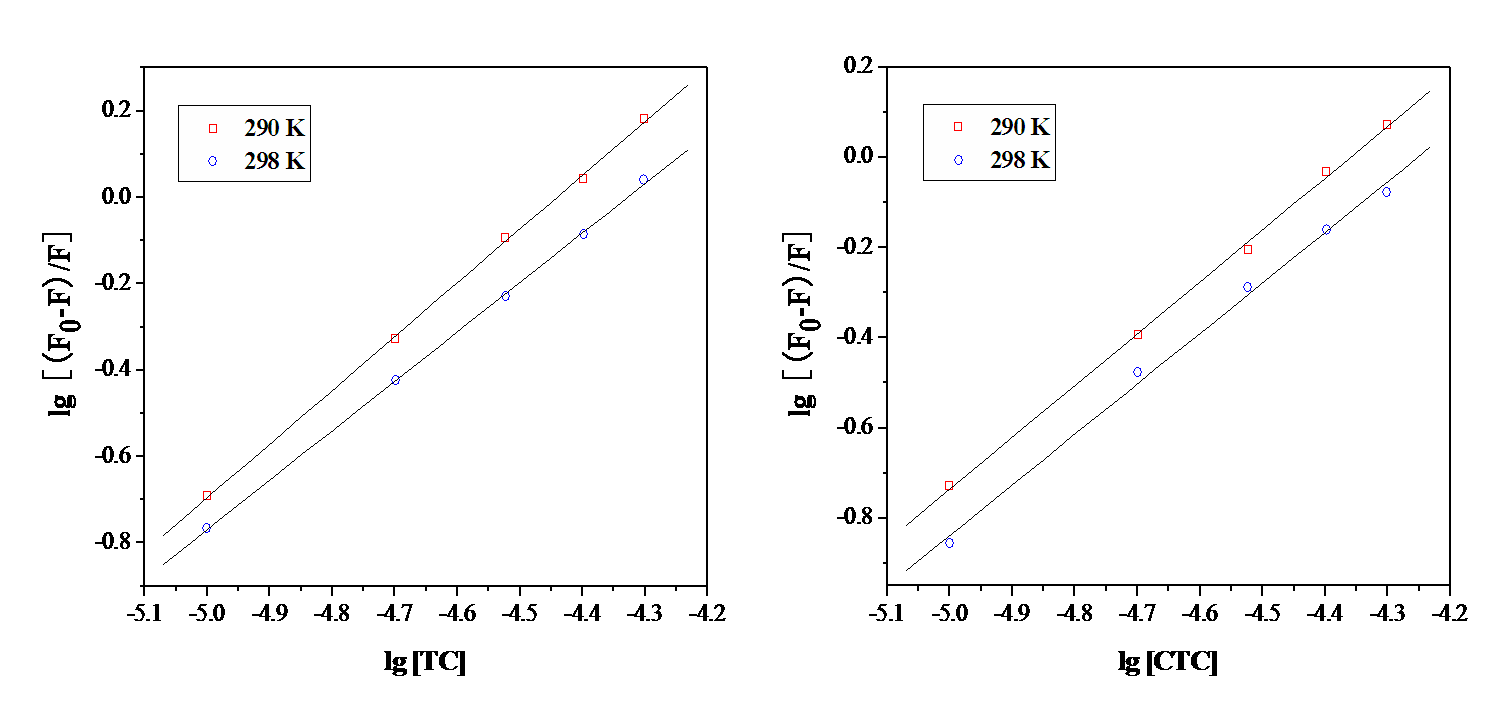

Supplement: Figure S2 — Plot of log [( F 0- F )/ F ] vs log [TC (CTC)] for the binding of TC and CTC to trypsin at various temperatures. (TIF) [file pone.0028361.s002.tif]

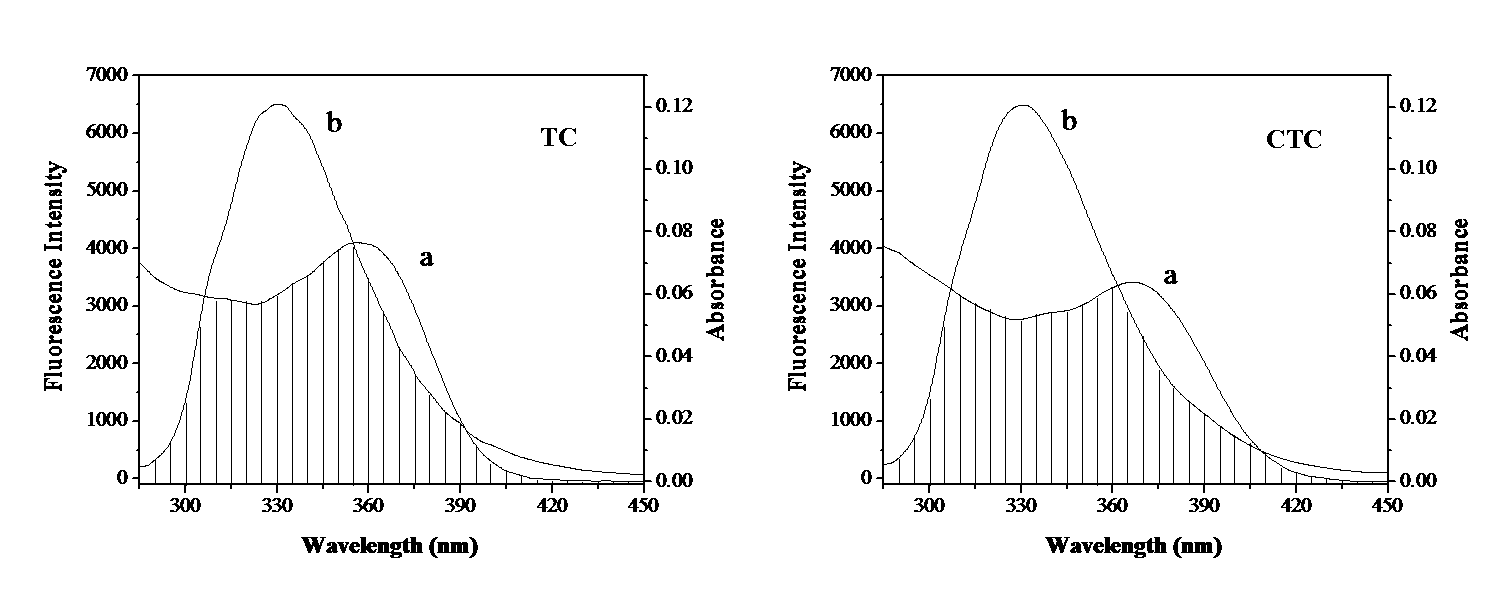

Supplement: Figure S3 — Overlap of the absorption spectrum of TC and CTC with fluorescence emission spectrum of trypsin (corrected). Conditions: Curve a: the absorption spectrum of TC (CTC); Curve b: the fluorescence emission spectrum of trypsin. The concentration of both TC (CTC) and trypsin are 5×10^−6^ mol L−1. (TIF) [file pone.0028361.s003.tif]
